# A clinical research on the potential pathogenesis of somatic cancer related cerebral venous sinus thrombosis

**DOI:** 10.1097/MD.0000000000015134

**Published:** 2019-05-13

**Authors:** Ziqiang Xian, Yicong Chen, Li Chen, Qiuhong Lu, Gelun Huang, Qixiong Qin, Jinsheng Zeng, Zhijian Liang

**Affiliations:** a Department of Neurology, The First Affiliated Hospital of Guangxi Medical University & Guangxi Key Laboratory Base of Precision Medicine in Cardio-cerebrovascular Diseases Control and Prevention & Guangxi Clinical Research Center for Cardio-cerebrovascular Diseases, Nanning, 530021, Guangxi; b Department of Neurology, The First Affiliated Hospital of Sun Yat-Sen University, Guangzhou, 510080, Guangdong, P.R. China.

**Keywords:** cerebral venous sinus thrombosis, pathogenesis, somatic solid cancer

## Abstract

To investigate the pathogenesis of somatic solid cancer-related cerebral venous sinus thrombosis (CVST).

A total of 174 patients with CVST were recruited from the hospital between January 2006 and December 2017 and divided into two groups: (1) somatic cancer-related CVST group, defined as active somatic solid cancer patients with acute CVST; (2) cancer group (CG), defined as active somatic solid cancer patients without CVST. The cancer group patients were age and gender-matched somatic cancer-related CVST group patients. In addition, the types and amount distribution of cancer in cancer group were also matched with somatic cancer-related CVST group patients.

Compared to cancer group patients, somatic cancer-related CVST group patients had more intracranial metastasis, a higher platelet count, higher plasma D-dimer, carcinoembryonic antigen (CEA) and cancer antigen (CA) 125 levels, a greater platelet to lymphocyte ratio (PLR), and a greater platelet to neutrophil ratio (PNR). The risk for CVST in somatic cancer-related CVST group patients increased independently by 0.7% (odds ratio [OR] 1.007; 95% confidence interval [CI] 1.000, 1.015; *P* = .047) with a 1 ng/ml increase in D-dimer levels, by 4.6% (OR 1.046; 95% CI 1.011, 1.083; *P* = .010) with a 1 U/ml increase in CEA, by 2.7% (OR 1.027; 95% CI 1.003, 1.051; *P* = .025) with a 1 U/ml increase in CA125, and by 10.6% (OR 1.106; 95% CI 1.002, 1.220; *P* = .045) with a 1 unit increase in PNR.

It was suggested that together impacts of elevated plasma D-dimer, CA125, CEA levels, and a greater PNR may lead to hypercoagulability and to trigger the development of cancer-related CVST.

## 1 Introduction

Cerebral venous sinus thrombosis (CVST) is an infrequent complication of patients with cancer. It was revealed that among all the patients with CVST, about 7% to 10% patients have cancer,^[1,2]^ and the incidence of CVST was about 0.3% to 4% in patients with cancer.^[3,4]^ CVST in cancer patients have been observed with various types of cancer, including somatic solid cancer such as lung cancer, ovarian cancer and breast cancer,^[5–7]^ hematological cancer such as lymphoma and leukaemia,^[8–10]^ and primary or metastatic cranial tumor.^[11–13]^ Moreover, recently it was demonstrated that the incidence of CVST in patients with cancer was higher than that of patients without cancer.^[14]^ In addition, some CVST patients with somatic solid cancer had not any conventional risks for CVST, such as infections of the orbit, mastoid or face, oral contraceptives, collagen diseases, pregnancy and the puerperium.^[5–7]^ As a result, it was suggested that some CVST in patients with cancer were caused by cancer which should be regarded as cancer-related CVST. Furthermore, previous studies showed that the pathogenesis of cancer-related CVST was involved in such several aspects as followed. First, the primary or metastatic intracranial tumors may obstruct the cerebral venous drainage and lead to CVST.^[11–13]^ Second, hematological cancers may lead to hypercoagulability directly, which in turn led to CVST.^[8–10]^ Finally, in except of intracranial metastatic,^[12,15]^ somatic solid cancers were speculated to lead to hypercoagulability indirectly and then lead to CVST.^[5–7,16]^ However, the clinical features and the pathogenesis of CVST in patients with somatic solid cancer remained to be elucidated. It was worth to explore what was the clinical features of patients with somatic solid cancers and what a role the somatic solid cancers play in development of CVST. In the present study, in order to explore the potential pathogenesis of somatic solid cancer-related CVST, the clinical data of CVST patients with somatic solid cancer from The First Affiliated Hospital of Guangxi Medical University and from The First Affiliated Hospital of Sun Yat-Sen University from January 2006 to December 2017 were collected and analyzed.

## 2 Material and methods

### 2.1 Subjects

This study was reviewed and approved by the First Affiliated Hospital of Guangxi Medical University Review Board and The First Affiliated Hospital of Sun Yat-Sen University Review Board. All patients were recruited from the First Affiliated Hospital of Guangxi Medical University and The First Affiliated Hospital of Sun Yat-Sen University from January 2006 to December 2017. The diagnosis of somatic cancer for all patients was pathologically confirmed. The diagnostic criteria for CVST were adopted according to the American Heart Association/American Stroke Association diagnostic criteria for CVST in 2011,^[17]^ that is, patients presenting with clinical symptoms related to increased intracranial pressure, such as headache and vomit, and signs of neurological deficits, such as limbs weakness and numbness, and mainly confirmed by computed tomography venography (CTV)/magnetic resonance venography (MRV)/digital subtraction angiography (DSA). A hemorrhagic complication or secondary subcortical infarct was revealed on intracranial MRI/CT.

In actuality, it is very difficult to identify somatic solid cancer-related CVST in modern clinical practice. In the present study, referring the definition of cancer-related stroke in previous studies,^[18–20]^ the somatic solid cancer-related CVST was defined as active somatic solid cancer patients with CVST without any conventional CVST risk factors such as pregnancy, oral contraceptive, and infection. The inclusion criteria for patients with somatic cancer-related CVST group:

(1) patients diagnosed with somatic solid cancer in the active phase with CVST without any conventional CVST risk factors;
(2) patients were firstly diagnosed with CVST without any conventional CVST risk factors, and were confirmed with active solid cancer during the hospitalization for anti-CVST therapy.

The exclusion criteria:

(1) patients with conventional risk factors for CVST;
(2) patients with hematologic malignancies such as leukemia and lymphoma;
(3) patients with disease other than somatic solid cancer which may trigger hypercoagulable state;
(4) CVST occurring more than five years after the somatic solid cancer diagnosis without the evidences of recurrence or metastasis;
(5) patients with general infection especially intracranial and facial suppurative infection.

The inclusion criteria for somatic cancer group: Patients with active somatic solid cancer alone. The age and sex in the cancer group patients were matched with somatic cancer-related CVST group patients. In addition, the types and amount distribution of cancer were also matched with somatic cancer-related CVST group patients. The exclusion criteria:

(1) patients with conventional CVST risk factors;
(2) patients with more than one type of somatic cancer;
(3) organ failure.

### 2.2 Collection of clinical data

The general demographic characteristics, as well as routine blood examinations and blood biochemical assays in two groups were collected. Data related to somatic cancer were collected, including pathological type, metastasis, grading, and staging; and treatment information, including radiotherapy, chemotherapy, and surgical resection; and tumor markers, such as cancer antigen (CA) 125, CA199, and carcinoembryonic antigen (CEA). Data related to CVST were also collected including risk factors such as pregnancy, oral contraceptive, infection, clinical manifestation, and biochemical test of CVST, such as full thrombotic workup, D-dimer level, cerebrospinal fluid examination, as well as imaging data such as MRV/CTV/DSA. All blood examinations and coagulation biochemical assays related to CVST were collected before anti-coagulant therapy. In order to avoid the impacts of cancer progress as possible, in 30th after CVST onset, a modified Rankin Scale (mRS) was used to evaluate the patients’ prognosis.

### 2.3 Statistical methods

All statistical analyses were performed using SPSS16.0 (Abacus Concepts, Inc, Chicago, IL). Independent sample *t*-tests were used for quantitative data and chi-square tests were used for qualitative data. Multivariable logistic regression analysis was performed to predict the independent contributions of factors in somatic cancer-related CVST group vs cancer group. Significant variables with *P* < .05 in univariate analyses were considered explanatory variables and were entered together into multivariable models. A *P* value < .05 was considered statistically significant.

## 3 Results

In the present study, according to the inclusion criteria, 30 somatic cancer-related CVST group patients with mean age (46.60 ± 10.53 years) were included, at the same time, 120 somatic cancer group patients with mean age (46.78 ± 10.48 years) were included as controls. The sex and age distributions were comparable among the two groups. As expected, no significant differences in age or gender were observed in these two groups. The demographic characteristics are listed in Table 1.

**Table 1:**
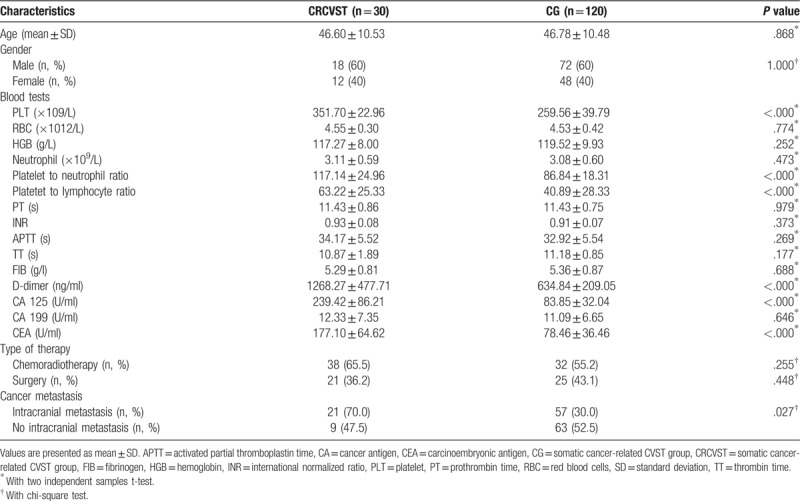
The clinical features of CRCVST compared to CG.

**Figure 1:**
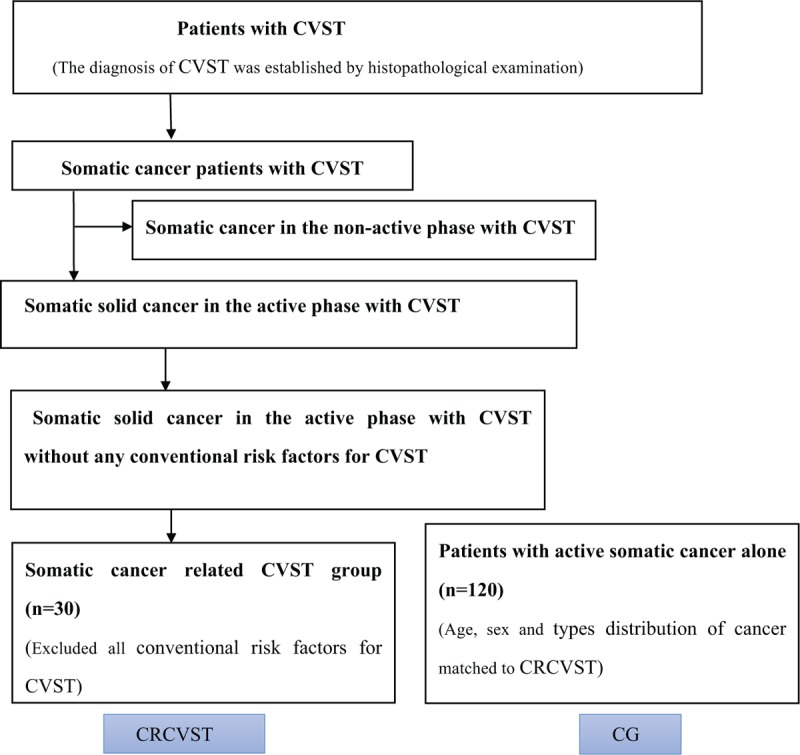
Patient selection. Thirty somatic cancer-related CVST patients were included as CRCVST. Meanwhile, 120 patients with active somatic cancer alone were recruited as control. Detailed information was analyzed and contrasted between two groups.

Among the somatic cancer-related CVST group patients, there were 8 kinds of cancers, the top 3 of which were lung cancer (9 cases, 31.00%), followed by breast cancer (7 cases, 23.33%), and gastric carcinoma (4 cases, 13.33%). In the somatic cancer-related CVST group patients, 24 out of 30 patients (80.00%) suffered from CVST within 4 months and 6 out of 30 patients (20.00%) suffered from CVST 4 to 12 months. Furthermore, there were 3 cases (10.00%) with CVST as the initial clinical manifestation, and the conceal cancers were confirmed during hospitalization (Fig. 1).

When the clinical characteristics of somatic cancer-related CVST group patients were compared to somatic cancer group patients, most blood biochemical assays and type of therapy were not significantly different. However, blood test endpoints, including plasma D-dimer, CEA, cancer antigen (CA) 125 levels, platelet (PLT), platelet to lymphocyte ratio (PLR), and platelet to neutrophil ratio (PNR) were significantly different between somatic cancer-related CVST group and CG. In addition, more somatic cancer-related CVST group patients had intracranial metastasis (Table 1).

To identify the risk factors for somatic cancer-related CVST, we analyzed seven significant variables, including intracranial metastasis, D-dimer, CA125, CEA, PLT, PLR, and PNR by multivariate logistic regression analysis. However, only D-dimer, CA125, CEA, and PNR entered the final models. The multiple model could be described using the following equation: logit *P* = ln(*P*/1 − *P*) = β0 + β1X1 + β2X2 + β3X3 + β4X4. The final regression equation was established as follows: logit *P* = –27.260 + 0.007X1 + 0.045X2 + 0.033X3 + 0.101X4. The risk of CVST in patients with somatic solid cancer increased independently by 0.7% (odds ratio [OR] 1.007; 95% confidence interval [CI] 1.000, 1.015; *P* = .047) with a 1 ng/ml increase in D-dimer levels, by 4.6% (OR 1.046; 95% CI 1.011, 1.083; *P* = .010) with a 1 U/ml increase in CEA, by 2.7% (OR 1.027; 95% CI 1.003, 1.051; *P* = .025) with a 1 U/ml increase in CA125, and by 10.6% (OR 1.106; 95% CI 1.002, 1.220; *P* = .045) with a 1 unit increase in PNR (Table 2).

**Table 2:**
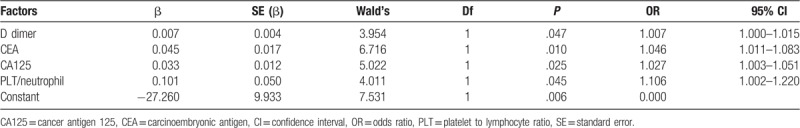
Multivariate logistic regression analysis.

## 4 Discussion

CVST is an infrequent but life-threatening complication in patients with cancer. Data of case reports on cancer patient with CVST have been documented for years, and many types of cancer have been reported to develop CVST.^[5–8]^ In order to understand the general clinical features and the risk for CVST in patients, recently Silvis and his colleagues ran a retrospective case-control study.^[14]^ It was found that the incidence of CVST of patients with cancer was higher than that of patients without cancer. Hematological cancer was the most frequent type of cancer, followed by breast cancer, gastrointestinal cancer, and lung cancer, and most of those patients developed CVST within the first year after the diagnosis of cancer. In the present study, among somatic cancer-related CVST group patients, lung cancer, breast cancer, and gastric carcinoma are the top three type of cancer, which was similar to the finding of the study mentioned above.^[14]^ Most cancer-related CVST patients develop CVST within 4 months after the diagnosis of cancer. Similar phenomenon that somatic cancer patients tend to develop CVST within 4 months after the diagnosis of cancer were found in previous study.^[4]^ It was suggested that as soon as somatic cancer was diagnosed, precautions to prevent cancer-related CVST should be taken into consideration. Additionally, CVST as the initial clinical manifestation in 3 patients were observed in the present study, which conceal cancers were firstly demonstrated during the hospitalization. CVST representing the initial manifestation of occult cancer had been found in previous studies.^[21–24]^ It was suggested that CVST patients with unknown pathogenesis should take measures to screen out concealing cancers.

As the incidence of CVST increased in patients with cancer, cancer was hypothesized to play a role in the development of CVST, which was known as cancer-related CVST. As it was proved in previous studies that the primary or metastatic intracranial tumors may lead to CVST by obstructing the cerebral venous drainage,^[11–13]^ and that hematological cancers may lead to CVST by causing hypercoagulability directly,^[8–10]^ the pathogenesis of somatic solid cancer-related CVST may be more complicated.

In the present study, there were two classification of patients with somatic cancer-related CVST group, of which one had intracranial metastatic, the other had not. The pathogenesis of somatic cancer-related CVST group in those patients with intracranial metastatic may be similar to the above study mentioned leading to CVST by obstructing the cerebral venous drainage.^[11–13]^ However, in the present study there were more patients with somatic cancer-related CVST group that had not intracranial metastatic. The pathogenesis of CVST in such patients remained unknown. In 2015, Reddy et al^[23]^ reported a patient of 68-year-old man with clear cell renal carcinoma that was initially hospitalized for superior sagittal sinus thrombosis without risk factors for CVST or intracranial metastasis. As elevated plasma D-dimer meaning a hypercoagulable state^[25]^ and with elevated plasma D-dimer, cancer-related hypercoagulability was suspected to be associated with the occurrence of CVST. Bruce Sigsbee et al^[26]^ prospectively recruited a total of 21 cancer patients with superior sagittal sinus thrombosis and found that 33.3% patients had no evidence of intracranial metastasis but had hypercoagulability features such as shorted thrombin time (TT), further indicating that cancer-related hypercoagulability played an important role in the pathogenesis of cancer-related CVST. In the present study, somatic cancer-related CVST group patients had elevated plasma D-dimer, and further multivariate logistic regression analysis revealed that elevated plasma D-dimer may be an independent risk factor for cancer-related CVST. Therefore, it was suggested that the elevated plasma D-dimer may also involve the occurrence of somatic cancer-related CVST group.

Furthermore, in the present study, it was showed that somatic cancer-related CVST group patients had elevated plasma cancer marker levels, including CA125 and CEA. Further multivariate logistic regression analysis revealed that the elevated levels of CA125 and CEA may independently increase the risk of somatic cancer-related CVST group. Mucin produced by cancer cells was suspected to contribute to cancer-related hypercoagulability and may indirectly lead to CVST;^[21]^ as previous animal experiments have demonstrated that mucins secreted by cancer cells could trigger the reciprocal activation of platelets through adhesion-dependent, bidirectional signaling in neutrophils and platelets, and then trigger the formation of microthromboembolism in the blood;^[27]^ and as CA125 and CEA are also mucins produced by cancer cells, it was suggested that cancer cells introduced mucinous substances, such as CA125 and CEA, into the bloodstream to cause the formation of microthromboembolism in the blood, which, in turn, led to hypercoagulability and triggered the development of cancer-related CVST. Based on the findings mentioned above, in the present study it was suggested that elevated plasma CA125 and CEA levels in patients with CVST may trigger the cancer-related CVST by causing the formation of microthromboembolism in the blood.

In addition, it was found that a greater PNR was found in somatic cancer-related CVST group patients and it also increased the risk for CVST independently. Previous studies showed that both a greater neutrophil to lymphocyte ratio and a greater PLR may predict a worse prognosis of cancer patients.^[28–30]^ However, as the impacts of greater neutrophil to lymphocyte ratio and PLR upon the development of CVST was unclear, and the relationship between PNR and CVST was not found in previous studies, it need further studies to reveal that what a role of a greater PNR in somatic cancer-related CVST group play on the pathogenesis of somatic cancer-related CVST group. Finally, previous studies proved that the pathogenesis of cancer-related hypercoagulability involved multiple pathways (for example, tissue factor expressed by tumor cells may directly cause hypercoagulability due to its role in the extrinsic coagulation pathway^[31–33]^). However, it was unclear whether such pathophysiologic pathways mentioned above involve the development of somatic cancer-related CVST group or not. Further studies were needed to investigate the pathogenesis of somatic solid cancer CVST. As a retrospective study, there was always a possibility of bias associated with inaccurate data collection and inability to control the setting. A future prospective study with a larger number of patients is needed to confirm our findings.

## 5 Conclusions

In summary, the results of the present study suggested that together impacts of elevated plasma D-dimer, CA125, CEA levels, and a greater PNR may lead to hypercoagulability and to trigger the development of cancer-related CVST.

## Author contributions

**Conceptualization:** Ziqiang Xian, Yicong Chen, Li Chen, Zhijian Liang.

**Data curation:** Ziqiang Xian, Yicong Chen, Qiuhong Lu, Gelun Huang, Zhijian Liang.

**Formal analysis:** Ziqiang Xian, Li Chen, Qiuhong Lu, Gelun Huang, Jinsheng Zeng.

**Funding acquisition:** Zhijian Liang.

**Investigation:** Ziqiang Xian, Qixiong Qin.

**Methodology:** Ziqiang Xian, Yicong Chen, Li Chen, Qiuhong Lu, Jinsheng Zeng.

**Project administration:** Zhijian Liang.

**Resources:** Ziqiang Xian, Yicong Chen, Jinsheng Zeng.

**Software:** Ziqiang Xian, Li Chen, Zhijian Liang.

**Supervision:** Li Chen.

**Validation:** Jinsheng Zeng.

**Visualization:** Zhijian Liang.

**Writing – original draft:** Ziqiang Xian.
